# Personal Influenza Vaccination and Willingness to Receive Vaccination in Community Pharmacies Among Pharmacists and Family Physicians in Northwestern Romania: A Cross-Sectional Study

**DOI:** 10.3390/healthcare14182969

**Published:** 2026-09-11

**Authors:** Adina Honoria Sabadoș, Daniela Rahotă, Teodor Traian Maghiar, Timea Claudia Ghitea, Laura Gratiela Vicas, Lucia Georgeta Daina

**Affiliations:** 1 Doctoral School of Biological and Biomedical Sciences, University of Oradea, 1 University Street, 410087 Oradea, Romania; b_adinahonoria@yahoo.com or sabados.adinahonoria@student.uoradea.ro; 2 Department of Morphological Disciplines, Faculty of Medicine and Pharmacy, University of Oradea, 410028 Oradea, Romania; dr.rahota@gmail.com (D.R.); uro_doruletul@yahoo.com (T.T.M.); 3 Pharmacy Department, Faculty of Medicine and Pharmacy, University of Oradea, 1 University Street, 410087 Oradea, Romania; laura.vicas@gmail.com; 4 Department of Psycho-Neurosciences and Recovery, Faculty of Medicine and Pharmacy, University of Oradea, 1 December Sq., 410081 Oradea, Romania; lucidaina@gmail.com

**Keywords:** influenza vaccines, vaccination, pharmacies, pharmacists, physicians, family, health personnel, attitude to health

## Abstract

**Highlights:**

**What are the main findings?**

- Family physicians reported substantially higher annual influenza vaccination uptake, whereas pharmacists showed markedly greater willingness to receive influenza vaccination in community pharmacies.
- Professional category showed the strongest independent association with high willingness to use pharmacy-based vaccination; awareness of the service and annual personal vaccination were also associated with the outcome.

**What are the implications of the main findings?**

- Personal influenza vaccination behaviour and acceptance of the vaccination setting were related but distinct dimensions in this sample; the formal interaction test did not confirm effect modification.
- Implementation strategies should be profession-specific, combining improved awareness, stronger pharmacist vaccination engagement, and clearer collaborative and organisational frameworks for family physicians.

**Abstract:**

**Background:** Community pharmacy-based influenza vaccination may improve access, but healthcare professionals may evaluate the service differently according to professional role, previous vaccination behaviour, and awareness. This study assessed the association between personal influenza vaccination behaviour and willingness to receive influenza vaccination in a community pharmacy among pharmacists and family physicians in Northwestern Romania. **Methods:** We analysed a cross-sectional, voluntary, non-probability convenience sample of 700 returned questionnaires from professionals active in six Northwestern Romanian counties. After exclusion of 22 blank records, 678 responses remained (340 pharmacists and 338 family physicians); one out-of-range willingness response was excluded from outcome analyses (n = 677). The study-specific anonymous questionnaire included demographic, professional, vaccination behaviour, awareness, and willingness items. Formal expert-panel content validation, pilot testing, test–retest reliability, construct validation, and a content-validity index were not documented; the primary outcome was a single Likert item, for which internal consistency is not estimable. Between 1 October 2025 and 30 April 2026, 761 questionnaires were distributed, and 700 were returned. After exclusion of 22 blank records, 678 completed questionnaires remained. Because recruitment was non-probability-based and a complete sampling frame was unavailable, the return proportion should not be interpreted as a population response rate. Ordinal comparisons used the Mann–Whitney U and the Kruskal–Wallis tests; high willingness (scores 4–5) was analysed with multivariable logistic regression. A 10-fold internal cross-validation and a sensitivity ordinal-logistic model were also performed. **Results:** Women comprised 85.5% of respondents. Annual influenza vaccination was more frequent among family physicians than pharmacists (71.9% vs. 32.6%), whereas mean willingness to be vaccinated in a pharmacy was higher among pharmacists (3.41 ± 1.40 vs. 2.08 ± 1.28). High willingness was reported by 201/339 pharmacists (59.3%) and 57/338 family physicians (16.9%). In the adjusted binary model, pharmacist profession (aOR 7.39, 95% CI 4.60–11.89), awareness of pharmacy vaccination (aOR 3.71, 95% CI 2.01–6.85), and annual personal vaccination (aOR 2.07, 95% CI 1.16–3.69) were associated with high willingness. Apparent discrimination was good (AUC 0.804), while mean 10-fold cross-validated AUC was 0.789. The profession × vaccination behaviour interaction was not statistically significant (likelihood-ratio *p* = 0.472). **Conclusions:** In this regional, non-probability sample, pharmacists were more willing than family physicians to receive influenza vaccination in a community pharmacy despite lower annual personal uptake. Professional category, service awareness, and annual vaccination were associated with willingness, but measurement validity, causality, and generalisability cannot be established. The findings support profession-specific implementation hypotheses and require confirmation with a formally validated instrument, transparent sampling, and multi-region prospective studies.

## 1. Introduction

Seasonal influenza remains a major cause of vaccine-preventable morbidity and mortality, particularly among older adults, people with chronic diseases, pregnant women, and immunocompromised patients. International public-health authorities recommend annual influenza vaccination because it reduces severe disease, hospitalisation, and influenza-related mortality and can indirectly protect vulnerable populations. Nevertheless, vaccination coverage among the general population and healthcare professionals remains below recommended targets in many settings [1,2,3].

Healthcare professionals influence vaccine confidence through their advice, professional behaviour, and communication with patients. Previous studies suggest that healthcare workers who accept vaccination themselves are more likely to recommend it, whereas hesitancy among healthcare workers may undermine public confidence. Personal vaccination behaviour is therefore relevant to professional credibility, but it does not by itself demonstrate that recommendations are made or followed [4,5,6].

Several healthcare systems have expanded the role of community pharmacists by authorising pharmacist-administered influenza vaccination. This model can improve access and convenience through extended opening hours and frequent patient contact without an appointment. It has therefore become an increasingly important component of some immunisation strategies [7,8].

Acceptance of pharmacist-administered vaccination remains heterogeneous among healthcare professionals. Existing research has focused more often on public attitudes, patient acceptance, or pharmacist experience than on the views of professionals from complementary roles. In particular, it remains uncertain whether personal influenza vaccination behaviour is associated with willingness to use pharmacy-based vaccination and whether this association is different for pharmacists and family physicians [9,10].

Acceptance of pharmacy-based vaccination may be related to awareness of the service, professional identity, previous vaccination behaviour, age, sex, experience, and perceptions of responsibility and safety. Identifying associations among these variables may help inform implementation strategies and interprofessional collaboration while avoiding assumptions of causality in a cross-sectional design [11].

Romania is a relevant setting because pharmacist-administered influenza vaccination has only recently entered routine practice and regional evidence remains limited. The present study was conducted in the North-West development region, represented in the database by Bihor, Cluj, Satu Mare, Maramureș, Bistrița-Năsăud, and Sălaj counties [12].

The primary objective was to evaluate the association between personal influenza vaccination behaviour and willingness to receive influenza vaccination in a community pharmacy among pharmacists and family physicians working in Northwestern Romania. The secondary objective was to describe demographic and professional correlates of high willingness, including professional category, awareness of pharmacy vaccination, age, sex, and professional experience.

We hypothesised that annual personal influenza vaccination would be positively associated with willingness to receive vaccination in a community pharmacy. We prespecified professional category as a potential effect modifier but interpreted this hypothesis only through the formal profession × vaccination behaviour interaction test.

## 2. Materials and Methods

### 2.1. Study Design and Participants

This cross-sectional observational study included pharmacists and family physicians working in Bihor, Cluj, Satu Mare, Maramureș, Bistrița-Năsăud, and Sălaj counties, which comprise Romania’s North-West development region. Data were collected between 1 October 2025 and 30 April 2026. Recruitment was voluntary and non-probability-based, consistent with a regional convenience sample.

A total of 761 questionnaires were distributed, of which 700 were returned, corresponding to a questionnaire return proportion of 92.0%. Twenty-two returned questionnaires contained no substantive data and were excluded. The final eligible sample therefore comprised 678 respondents: 340 pharmacists and 338 family physicians. The professional category labels recorded in the database as ‘Farmacist’, ‘Medic de familie’, and ‘Medic familie’ were harmonised before analysis.

Because a complete sampling frame of all eligible pharmacists and family physicians in the six counties was not available, the 92.0% figure represents the return proportion among distributed questionnaires and not a population response rate. Selection bias cannot be quantified, and the sample should not be considered representative of all Romanian pharmacists or family physicians.

### 2.2. Questionnaire

The questionnaire was developed specifically for this study and covered demographic characteristics, professional background, personal influenza vaccination behaviour, awareness of pharmacy-based vaccination, willingness to use the service, and perceptions of safety, responsibility, barriers, and implementation. The supplied files preserve the item wording and response options but do not include a literature-to-item development matrix, expert-panel content validation, cognitive interviewing or pilot testing, test–retest assessment, construct-validity analysis, or a content-validity index. The instrument is therefore treated as an exploratory, study-specific questionnaire, not as a psychometrically validated scale.

Demographic variables included age, sex, county of professional activity, and years of professional experience. The database contains categorical responses and several spelling variants (for example, hyphen versus en-dash age and experience categories), which were harmonised before analysis.

Personal influenza vaccination behaviour was assessed using three recorded categories: annual vaccination, occasional vaccination, and never vaccinated.

Awareness of pharmacy-based vaccination was assessed with a dichotomous item asking whether respondents knew that influenza vaccination could be performed in community pharmacies.

The primary outcome was willingness to receive influenza vaccination in a community pharmacy, measured with one five-point Likert item ranging from 1 (not willing at all) to 5 (very willing). For the prespecified binary sensitivity analysis, scores 1–3 were classified as low willingness and scores 4–5 as high willingness. Because the primary outcome is a single item, internal consistency reliability (for example, Cronbach’s alpha) is not estimable and was not calculated. No test–retest or construct-validity evidence was available in the supplied files; no factor analysis or composite score reliability analysis was therefore performed. The binary cut-off was used only for an interpretable sensitivity contrast and is not a validated clinical threshold.

Questionnaire items were drafted following a review of the literature concerning influenza vaccination behaviour, acceptance of pharmacist-administered vaccination, perceived safety, professional responsibility, implementation barriers, and interprofessional collaboration. The items were subsequently adapted to the Romanian community pharmacy context. However, no formal literature-to-item matrix was developed, and the instrument did not undergo expert-panel content validation, cognitive interviewing, pilot testing, test–retest assessment, or construct-validity analysis.

### 2.3. Variables Included in the Analysis

The primary estimand was the association between personal influenza vaccination behaviour and willingness to receive influenza vaccination in a community pharmacy. The regression outcome was high willingness (score 4–5), with the original five-level willingness score retained for descriptive and sensitivity analyses.

The dependent variable for the logistic regression model was high willingness to receive influenza vaccination in a pharmacy (score 4–5).

Independent variables included professional category, personal influenza vaccination behaviour, awareness of pharmacy vaccination, age category, sex, and professional experience. Professional category was the principal comparison variable; the remaining variables were selected a priori as potential confounders or correlates.

- professional category (pharmacist or family physician);
- personal influenza vaccination behaviour;
- awareness of pharmacy-based vaccination services;
- age category;
- sex;
- professional experience.

Professional category was selected because pharmacists and family physicians have complementary roles in influenza prevention but may have different perspectives on pharmacy-based delivery. This choice does not imply that professional category causes willingness.

### 2.4. Statistical Analysis

All analyses were performed in Python 3.12 (Python Software Foundation, Wilmington, DE, USA) using pandas 2.2.3, SciPy 1.14.1, statsmodels 0.14.4, scikit-learn 1.5.2, and standard plotting libraries. The software and package versions were recorded as part of the analytical documentation to support reproducibility.

Continuous or ordinal scores were summarised as mean ± standard deviation and median with interquartile range; categorical variables were summarised as counts and percentages. Percentages were calculated using the relevant non-missing denominator.

Pearson chi-square tests were used for categorical comparisons between professional groups. Because the willingness item was ordinal and non-normally distributed, the Mann–Whitney U tests were used for two-group comparisons and the Kruskal–Wallis tests for comparisons across three vaccination behaviour categories.

The five-level willingness score was treated as ordinal rather than continuous. High willingness (4–5) was analysed additionally with chi-square tests and binary logistic regression, but the binary model was not treated as the only or definitive representation of the outcome.

High willingness (scores 4–5) was additionally analysed as a binary outcome using chi-square tests.

A multivariable binary logistic regression model estimated associations with high willingness (score 4–5) among 677 participants with valid outcome data. The model included profession, personal vaccination behaviour, awareness, age, sex, and professional experience.

Adjusted odds ratios (aORs), 95% confidence intervals (CIs), and two-sided *p*-values were reported. Wide CIs were interpreted as evidence of imprecision, particularly for small age and experience subgroups.

Apparent discrimination was evaluated with the area under the receiver operating characteristic curve (AUC), calibration with the Hosmer–Lemeshow goodness-of-fit test, and explained variation with the Nagelkerke pseudo-R^2^. To assess optimism and possible over-fitting, stratified 10-fold cross-validation was performed; the mean cross-validated AUC was compared with the apparent AUC. No external validation cohort was available.

Potential effect modification by professional category was evaluated by adding a profession × personal vaccination behaviour interaction term. The likelihood-ratio test compared the interaction model with the parsimonious model. Collinearity between age, professional experience, and other model variables was assessed using variance-inflation factors (VIFs); the largest observed VIF was approximately 5.3 for an age category indicator, indicating moderate rather than negligible overlap.

A two-sided *p*-value < 0.05 was considered statistically significant. Because the study was exploratory and based on a non-probability sample, *p*-values were interpreted together with effect sizes, confidence intervals, subgroup sizes, and the sampling limitations.

### 2.5. Data Quality

Before analysis, the dataset underwent a reproducible quality-control procedure: blank records were excluded; spelling variants for professional, age, and experience categories were harmonised; numeric fields were checked against their permitted ranges; and the single willingness value of 6 was treated as out-of-range and excluded from analyses involving willingness.

Complete-case data were used for the regression analyses. Thus, 677 participants contributed to models involving willingness. The same denominator is used in the revised tables and text; the 678-person denominator is retained for variables available for all eligible respondents.

The present analysis focused on the prespecified exposure (personal vaccination behaviour), the awareness item, and the willingness item. Other questionnaire fields were not incorporated into the primary model because a validated composite score, prespecified domain structure, and complete coding dictionary were not available. Personal vaccination behaviour was coded as 0 = never, 1 = occasional, and 2 = annual; awareness as 0 = unaware and 1 = aware; and willingness retained its original 1–5 ordering. No weighting, imputation, or data-driven reclassification was applied.

The reconstructed workflow was: anonymous questionnaire completion after information and consent; export to a tabular dataset; removal of blank records; harmonisation of professional, age, and experience labels; range checks; descriptive analysis of eligible records; and complete-case regression using valid 1–5 willingness responses. This describes data processing and does not establish how participants were initially contacted.

### 2.6. Ethical Considerations

The study was conducted in accordance with the Declaration of Helsinki and was approved by the Ethical Committee of the Faculty of Medicine and Pharmacy, University of Oradea (approval code 37, 30 September 2025, Oradea, Romania).

Participation was voluntary and anonymous, and no personally identifiable information was collected. Informed consent was obtained before questionnaire completion.

The study protocol received approval from the appropriate institutional ethics committee of the University of Oradea (approval code 37, dated 30 September 2025).

## 3. Results

### 3.1. Participant Characteristics and Personal Influenza Vaccination Behaviour

Of 700 database records, 22 were blank. The analytical sample comprised 678 professionals: 340 pharmacists and 338 family physicians. One willingness value of 6 was outside the prespecified 1–5 range and was excluded from willingness analyses, leaving 677 valid outcome records.

The near-equal group sizes are a feature of the returned sample, not evidence of representative sampling. County distribution and participant characteristics differed between professional groups; these differences are shown in Table 1 and should be considered when interpreting crude comparisons.

Women comprised 580/678 participants (85.5%). More than 20 years of experience was reported by 43.1% overall and was more frequent among family physicians, while 5–10 years of experience was more frequent among pharmacists. Bihor accounted for 54.9% of the sample; the remaining respondents were distributed across Cluj, Satu Mare, Maramureș, Bistrița-Năsăud, and Sălaj.

Annual influenza vaccination was reported by 354/678 participants (52.2%), occasional vaccination by 235 (34.7%), and no previous vaccination by 89 (13.1%). Annual vaccination was more common among family physicians than pharmacists (71.9% vs. 32.6%; *p* < 0.001).

Among 677 valid willingness responses, pharmacists had a higher mean score than family physicians (3.41 ± 1.40 vs. 2.08 ± 1.28; *p* < 0.001). High willingness (score 4–5) was reported by 201/339 pharmacists (59.3%) and 57/338 family physicians (16.9%) for 258/677 participants (38.1%).

**Figure 1:**
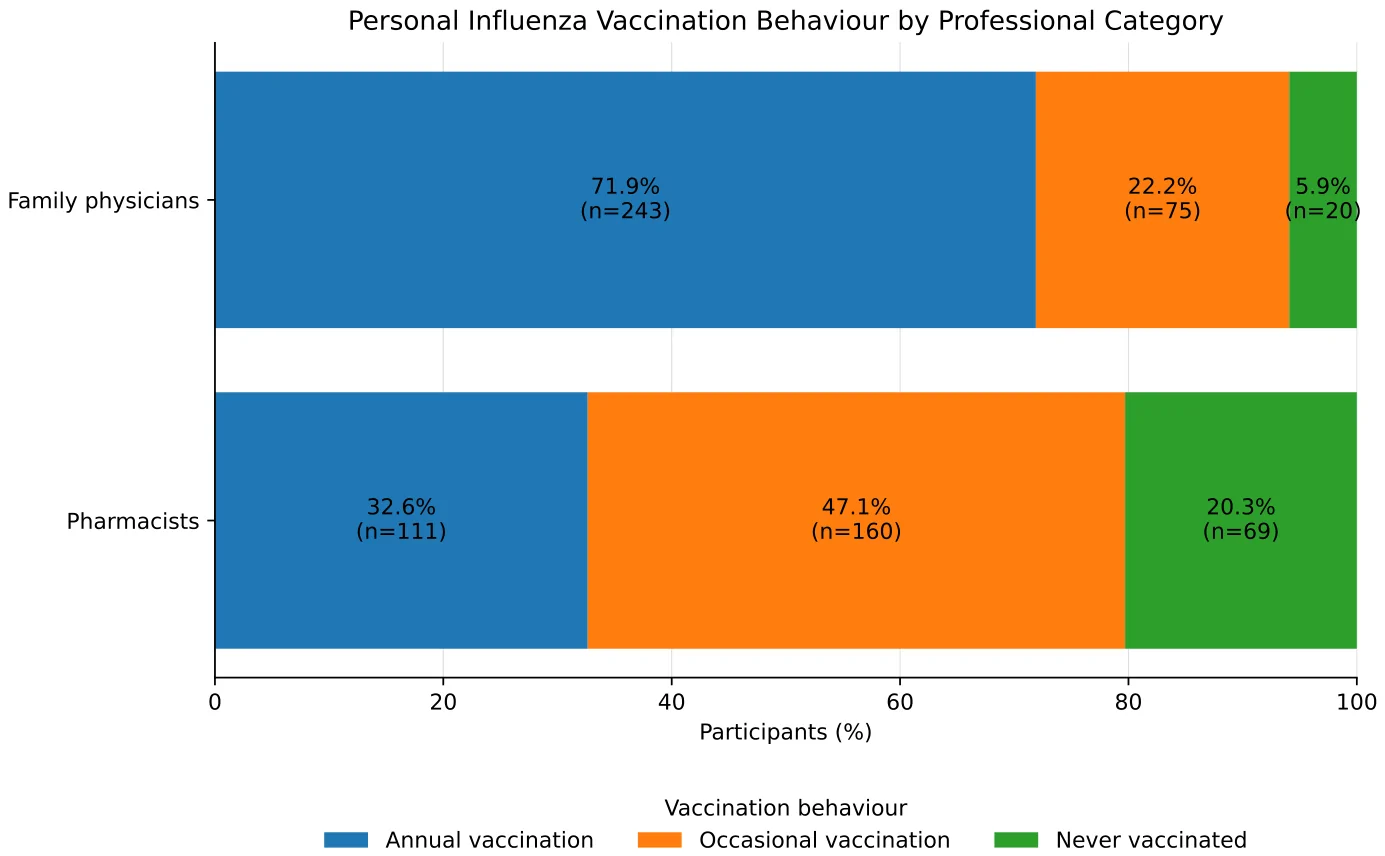
Personal influenza vaccination behaviour among pharmacists and family physicians. Bars represent the percentage distribution within each professional category; absolute frequencies are presented in parentheses. Annual influenza vaccination was reported by 32.6% of pharmacists and 71.9% of family physicians, whereas 20.3% and 5.9%, respectively, had never received an influenza vaccine.

Thus, the sample showed a contrast between past vaccination behaviour and acceptance of the pharmacy setting: family physicians reported higher annual uptake, whereas pharmacists reported greater willingness. Because the groups differed at baseline and were not sampled probabilistically, this contrast is descriptive and not causal (Figure 1).

### 3.2. Association Between Personal Influenza Vaccination Behaviour and Acceptance of Pharmacy-Based Vaccination

Personal vaccination behaviour was available for all 678 eligible participants; willingness was valid for 677. In the unadjusted analysis, willingness differed across vaccination categories (Kruskal–Wallis H = 13.72, *p* = 0.001), and high willingness was 31.7% among annual, 48.1% among occasional, and 37.1% among never vaccinated respondents (χ^2^ = 16.05, *p* < 0.001).

**Figure 2:**
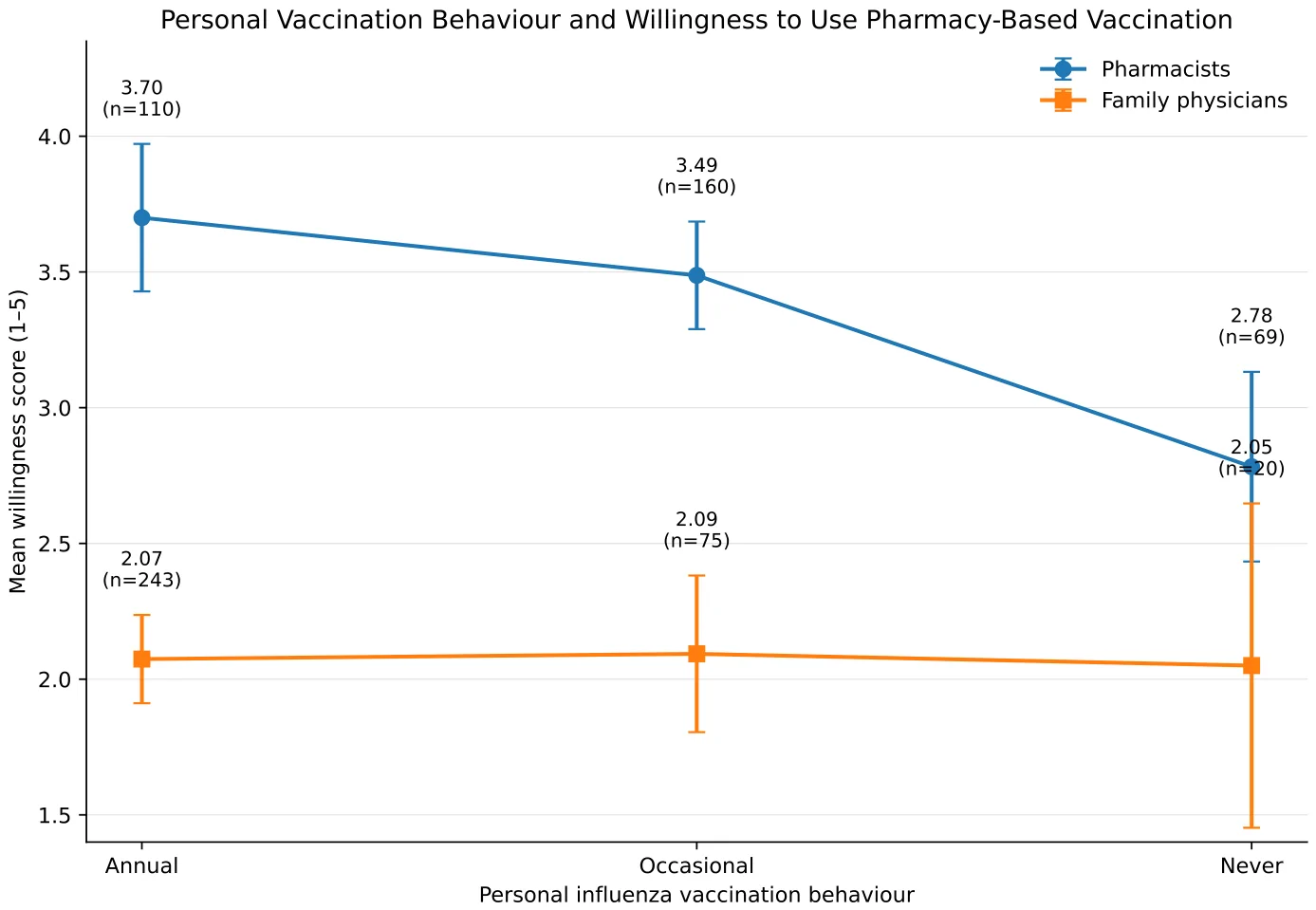
Association between personal influenza vaccination behaviour and willingness to receive influenza vaccination in a community pharmacy, stratified by professional category. Points represent mean willingness scores, and error bars indicate 95% confidence intervals. Among pharmacists, willingness was highest in those vaccinated annually and lowest in those who had never been vaccinated. Among family physicians, willingness remained consistently low across all personal vaccination categories. One out-of-range response was excluded.

The overall ordering was influenced by professional composition: family physicians were concentrated in the annual vaccination group but had low willingness to use a pharmacy. Within pharmacists, annual vaccination was associated with higher willingness than never vaccination (mean 3.70 vs. 2.78; high willingness 65.5% vs. 42.0%; *p* < 0.01), whereas no corresponding difference was detected among family physicians (Table 2 and Figure 2).

These stratified comparisons are exploratory. The smallest subgroups and the single invalid willingness response reduce precision, and the formal profession × vaccination behaviour interaction test is reported in Section 3.3.

### 3.3. Multivariable Analysis of Factors Associated with Willingness to Receive Influenza Vaccination in Community Pharmacies

A multivariable logistic regression model examined correlates of high willingness (score 4–5) among 677 complete cases. The model included profession, personal vaccination behaviour, awareness, age, sex, and professional experience. The model was statistically different from the intercept-only model (likelihood-ratio *p* < 0.001; Nagelkerke R^2^ = 0.345).

The largest adjusted association was observed for pharmacist profession versus family physician (aOR 7.39, 95% CI 4.60–11.89). Awareness of pharmacy vaccination (aOR 3.71, 95% CI 2.01–6.85) and annual personal vaccination (aOR 2.07, 95% CI 1.16–3.69) were also associated with high willingness; occasional vaccination was not clearly different from never vaccination (aOR 1.68, 95% CI 0.95–2.97).

Age and experience estimates were non-monotonic and imprecise in several categories. The female-versus-male estimate was also uncertain because men represented only 14.5% of the sample. These coefficients should be read as conditional associations within this dataset, not as transferable predictors.

The apparent AUC was 0.804, while the mean internally cross-validated AUC was 0.789; no external validation was performed. Calibration was acceptable by the Hosmer–Lemeshow test, but this does not establish transportability.

Age and professional experience showed moderate overlap (maximum VIF approximately 5.3), so coefficient stability remains a concern. The ordinal-logistic sensitivity analysis produced broadly similar directions for profession, awareness, and annual vaccination.

**Figure 3:**
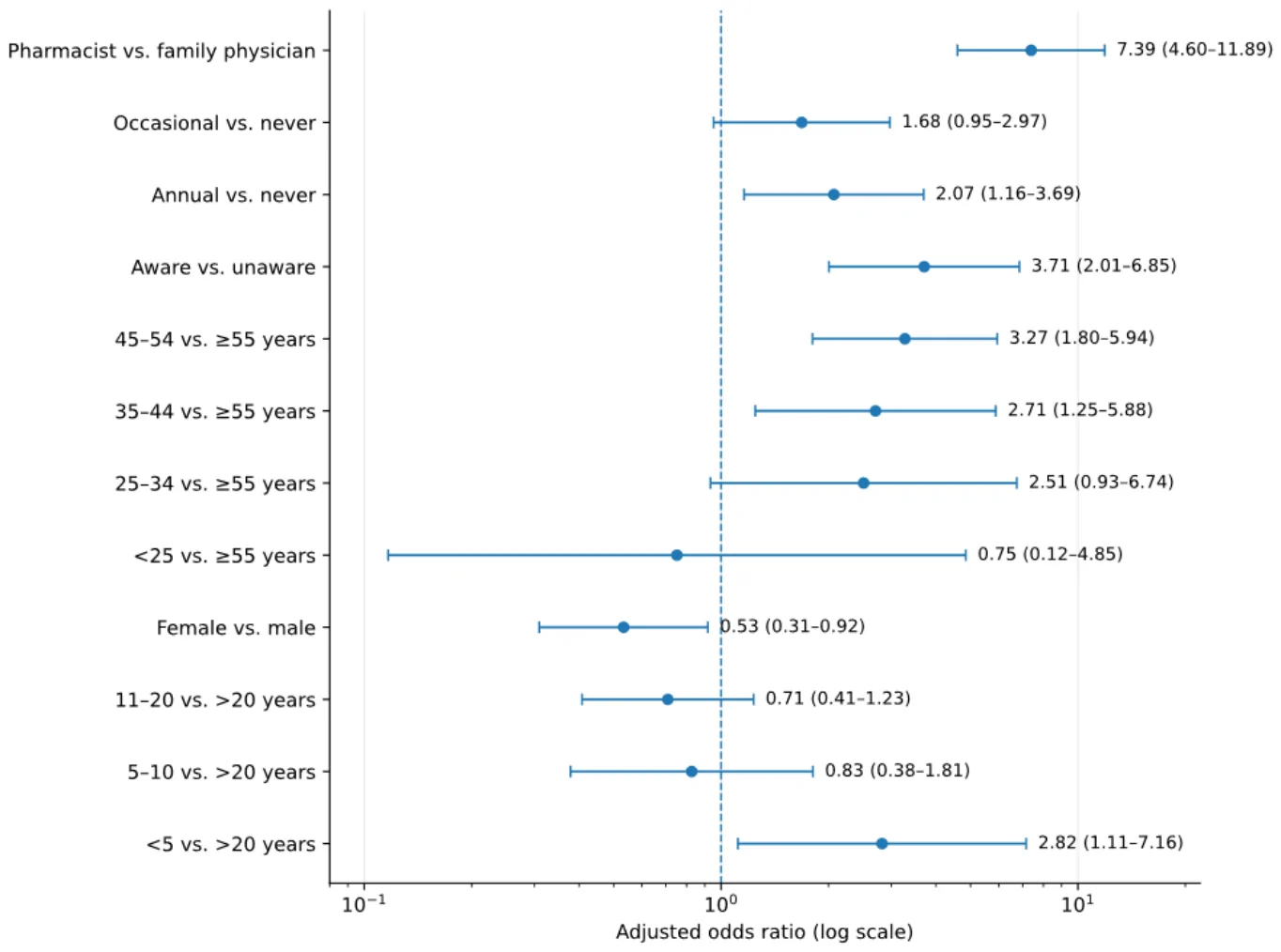
Multivariable analysis of factors associated with willingness to receive influenza vaccination in a community pharmacy. Points represent adjusted odds ratios, and horizontal lines indicate 95% confidence intervals. High willingness was defined as a score of 4 or 5. The model was adjusted for profession, personal influenza vaccination behaviour, awareness of pharmacy-based vaccination, age, sex, and professional experience. The vertical dashed line indicates the null value of OR = 1.

The profession × vaccination behaviour interaction did not improve model fit (likelihood-ratio χ^2^ = 1.50, df = 2, *p* = 0.472). The parsimonious model was retained; the pharmacist-specific pattern is therefore exploratory rather than evidence of effect modification (Table 3 and Figure 3).

Among 677 participants with valid awareness and willingness data, 565 (83.5%) were aware of pharmacy-based vaccination, and 112 (16.5%) were unaware. Mean willingness was 2.88 ± 1.51 among aware respondents versus 2.05 ± 1.21 among unaware respondents; high willingness was 42.7% versus 15.2%.

Awareness remained associated with high willingness in the adjusted model (aOR 3.71, 95% CI 2.01–6.85), but both variables were measured at the same time. The direction of association is therefore uncertain, and the result should not be interpreted as evidence that an awareness campaign would necessarily increase acceptance (Table 4).

## 4. Discussion

The principal finding was a contrast between professions: family physicians reported higher annual influenza vaccination, whereas pharmacists reported greater willingness to use a community pharmacy for vaccination. These are related but distinct dimensions, and neither was measured with a formally validated instrument.

The professional contrast should be interpreted as a descriptive association in a voluntary, non-probability sample. The large adjusted profession coefficient may reflect baseline imbalance, self-selection, and unmeasured organisational or legal concerns rather than a causal effect of professional identity.

The observed patterns are compatible with several explanations, including differences in professional role, responsibility, documentation, safety expectations, and familiarity with pharmacy-based vaccination. These mechanisms were not directly measured and should remain hypotheses for future studies [13,14,15,16,17,18,19].

Annual vaccination remained associated with high willingness after adjustment, while occasional vaccination did not. This may reflect shared health orientation or confidence, but cross-sectional data cannot establish temporal order or causality [16].

Awareness was associated with higher willingness, but awareness and willingness were assessed concurrently. Information strategies should therefore be paired with clear legal, safety, documentation, referral, and responsibility frameworks and evaluated prospectively [8,20,21,22,23,24,25].

Age, sex, and professional experience showed less stable associations. The high proportion of women, moderate age–experience overlap, and small subgroups limit interpretation and make generalisation to other professional populations inappropriate.

The formal profession × vaccination behaviour interaction was not statistically significant (*p* = 0.472). The pharmacist-specific pattern should therefore be treated as exploratory and tested in a larger, prospectively sampled study.

Model discrimination was good in the development data but lower after internal cross-validation, with no external validation. The model is consequently an explanatory association model, not a clinical prediction rule [26,27,28,29,30].

The model showed an apparent AUC of 0.804 in the development dataset and a mean internally cross-validated AUC of 0.789. However, these estimates do not establish external validity or compensate for the sampling and measurement limitations.

For implementation, pharmacists may need training and delivery safeguards, whereas family physicians may need clear referral pathways, shared documentation, adverse-event responsibility, and interprofessional communication. These recommendations are hypothesis-generating and were not tested by the present survey [31,32].

### 4.1. Implications for Practice and Policy

Implementation strategies should therefore be profession-specific and evaluated prospectively. For pharmacists, training should be paired with safe-delivery protocols; for family physicians, implementation should clarify referral, documentation, adverse-event responsibility, continuity of care, and interprofessional communication.

### 4.2. Strengths and Limitations

The nearly balanced professional groups and the availability of vaccination behaviour, awareness, willingness, and professional characteristics in one record enabled direct descriptive comparison. The analysis also included stratified comparisons, an ordinal sensitivity model, and internal cross-validation.

The limitations nevertheless materially constrain credibility and interpretation. First, recruitment was voluntary and non-probability-based, and a complete sampling frame of eligible professionals was not available. Although 761 questionnaires were distributed and 700 were returned, the resulting 92.0% represents a return proportion among distributed questionnaires rather than a population response rate. Selection bias cannot be quantified, and the six-county sample should not be generalised to all Romanian healthcare professionals.

Second, the cross-sectional design and self-reported measures prevent causal inference. Willingness is an intention, not observed service uptake, and no non-healthcare comparison group was available.

Third, the questionnaire was developed for this study, and the supplied files contain no documentation of expert-panel content validation, pilot testing, test–retest reliability, construct validity, or a content-validity index. The primary outcome was a single Likert item, so internal consistency reliability is not estimable. No factor analysis or composite score reliability analysis was appropriate for the primary outcome. This psychometric gap reduces confidence in measurement validity and limits generalisability; it should be addressed through formal instrument development before the findings are used to support implementation decisions.

Fourth, the groups differed in sex, age, experience, county distribution, and personal vaccination behaviour, and some subgroups were small. Adjustment cannot remove bias from unmeasured differences or selection into the sample.

Finally, dichotomising the five-level outcome loses information and depends on the chosen cut-off. Ordinal analyses were retained as a sensitivity check, but neither internal validation nor statistical adjustment can compensate for uncertain sampling and measurement validity. Future work should use an enumerated sampling frame, report invited and responding professionals by stratum, complete formal questionnaire validation, and evaluate actual vaccination uptake prospectively across multiple regions.

## 5. Conclusions

In this voluntary, non-probability sample from six counties in Northwestern Romania, pharmacists reported lower annual influenza vaccination but greater willingness to receive vaccination in a community pharmacy than family physicians. Awareness and annual vaccination were associated with high willingness, but the profession × vaccination behaviour interaction was not statistically significant.

These findings are exploratory associations, not causal effects, validated predictions, or population estimates. Past vaccination behaviour and acceptance of the pharmacy setting should not be treated as interchangeable.

Before implementation decisions are based on these results, the questionnaire should undergo expert content review, pilot testing, test–retest assessment, and construct validation, and the study should be repeated using transparent probability-based or enumerated stratified sampling across multiple regions with prospective measurement of actual service uptake.

## Figures and Tables

**Table 1:** Baseline demographic characteristics and personal influenza vaccination behaviour among pharmacists and family physicians. Willingness analyses use 677 valid responses because one out-of-range value (6) was excluded; therefore, the willingness denominators are shown explicitly.

Characteristic	Pharmacists (n = 340)	Family Physicians (n = 338)	Total (n = 678)	*p*-Value
Female sex, n (%)	310 (91.2)	270 (79.9)	580 (85.5)	<0.001
Male sex, n (%)	30 (8.8)	68 (20.1)	98 (14.5)	
Age categories, n (%)				
<25 years	8 (2.4)	0 (0.0)	8 (1.2)	<0.001
25–34 years	101 (29.7)	32 (9.5)	133 (19.6)	
35–44 years	99 (29.1)	56 (16.6)	155 (22.9)	
45–54 years	105 (30.9)	98 (29.0)	203 (29.9)	
≥55 years	27 (7.9)	152 (45.0)	179 (26.4)	
Personal influenza vaccination behaviour, n (%)				
Annual influenza vaccination	111 (32.6)	243 (71.9)	354 (52.2)	<0.001
Occasional vaccination	160 (47.1)	75 (22.2)	235 (34.7)	
Never vaccinated	69 (20.3)	20 (5.9)	89 (13.1)	
Willingness to receive influenza vaccination in a pharmacy (1–5), mean ± SD	3.41 ± 1.40 (n = 339)	2.08 ± 1.28 (n = 338)	2.75 ± 1.50 (n = 677)	<0.001
High willingness (score 4–5), n (%)	201 (59.3%)	57 (16.9%)	258 (38.1%)	<0.001

**Table 2:** Willingness to receive influenza vaccination in a community pharmacy according to personal vaccination behaviour and professional category.

Professional Group	Personal Vaccination Behaviour	n	Mean ± SD	Median (IQR)	High Willingness, n (%)	*p*-Value, Ordinal Score	*p*-Value, High Willingness
Overall	Annual vaccination	353	2.58 ± 1.53	2 (1–4)	112 (31.7)	0.001	<0.001
	Occasional vaccination	235	3.04 ± 1.42	3 (2–4)	113 (48.1)		
	Never vaccinated	89	2.62 ± 1.44	2 (1–4)	33 (37.1)		
Pharmacists	Annual vaccination	110	3.70 ± 1.44	4 (3–5)	72 (65.5)	<0.001	0.004
	Occasional vaccination	160	3.49 ± 1.27	4 (3–4)	100 (62.5)		
	Never vaccinated	69	2.78 ± 1.45	3 (1–4)	29 (42.0)		
Family physicians	Annual vaccination	243	2.07 ± 1.29	2 (1–3)	40 (16.5)	0.974	0.914
	Occasional vaccination	75	2.09 ± 1.25	2 (1–3)	13 (17.3)		
	Never vaccinated	20	2.05 ± 1.28	2 (1–2)	4 (20.0)		

High willingness was defined as a score of 4 or 5. Ordinal comparisons used the Kruskal–Wallis test, and high willingness comparisons used the chi-square test. The annual vaccination overall row has n = 353 because one annual vaccination respondent had an out-of-range willingness value.

**Table 3:** Multivariable logistic regression analysis.

Variable	Adjusted OR	95% CI	*p*-Value
Pharmacist vs. family physician	7.39	4.60–11.89	<0.001
Occasional vaccination vs. never	1.68	0.95–2.97	0.073
Annual vaccination vs. never	2.07	1.16–3.69	0.014
Aware vs. unaware of pharmacy vaccination	3.71	2.01–6.85	<0.001
Age 45–54 vs. ≥55 years	3.27	1.80–5.94	<0.001
Age 35–44 vs. ≥55 years	2.71	1.25–5.88	0.012
Age 25–34 vs. ≥55 years	2.51	0.93–6.74	0.068
Age <25 vs. ≥55 years	0.75	0.12–4.85	0.764
Female vs. male	0.53	0.31–0.92	0.023
Experience 11–20 vs. >20 years	0.71	0.41–1.23	0.224
Experience 5–10 vs. >20 years	0.83	0.38–1.81	0.635
Experience <5 vs. >20 years	2.82	1.11–7.16	0.029

Model performance: Nagelkerke R^2^ = 0.345; apparent AUC = 0.804, 95% bootstrap CI approximately 0.770–0.836; mean stratified 10-fold cross-validated AUC = 0.789; Hosmer–Lemeshow χ^2^ = 6.10, df = 8, *p* = 0.636. VIFs indicated moderate overlap between age and professional experience (maximum approximately 5.3).

**Table 4:** Awareness of pharmacy-based vaccination services and willingness to receive influenza vaccination in a community pharmacy.

Awareness	n	Mean ± SD	Median	High Willingness (4–5), n (%)
Unaware	112	2.05 ± 1.21	2	17 (15.2)
Aware	565	2.88 ± 1.51	3	241 (42.7)

Statistical analysis: Mann–Whitney U test, χ^2^ test for high willingness. Logistic regression reference: adjusted OR = 3.71 (95% CI 2.01–6.85), *p* < 0.001.

## Data Availability

The original contributions presented in this study are included in this article. Further inquiries can be directed to the first authors.

## Abbreviations

aOR	Adjusted Odds Ratio
AUC	Area Under the Receiver Operating Characteristic Curve
CI	Confidence Interval
IQR	Interquartile Range
OR	Odds Ratio
ROC	Receiver Operating Characteristic
SD	Standard Deviation
